# Validation of Oura ring energy expenditure and steps in laboratory and free-living

**DOI:** 10.1186/s12874-023-01868-x

**Published:** 2023-02-24

**Authors:** Emilia Kristiansson, Jonatan Fridolfsson, Daniel Arvidsson, Agneta Holmäng, Mats Börjesson, Ulrika Andersson-Hall

**Affiliations:** 1 Center for Health and Performance, Department of Food and Nutrition, and Sport Science Faculty of Education, University of Gothenburg, Gothenburg, Sweden; 2 Institute of Neuroscience and Physiology, Department of Physiology, Sahlgrenska Academy, University of Gothenburg, Gothenburg, Sweden; 3 Department of Molecular and Clinical Medicine, Institute of Medicine, Sahlgrenska Academy, University of Gothenburg, Gothenburg, Sweden; 4 Sahlgrenska University Hospital, Region Västra Götaland, Gothenburg, Sweden

**Keywords:** Oura smart ring, Indirect calorimetry, Wearable devices, Energy expenditure, Step count, Validation, Accelerometer

## Abstract

**Background:**

Commercial activity trackers are increasingly used in research and compared with research-based accelerometers are often less intrusive, cheaper, with improved storage and battery capacity, although typically less validated. The present study aimed to determine the validity of Oura Ring step-count and energy expenditure (EE) in both laboratory and free-living.

**Methods:**

Oura Ring EE was compared against indirect calorimetry in the laboratory, followed by a 14-day free-living study with 32 participants wearing an Oura Ring and reference monitors (three accelerometers positioned at hip, thigh, and wrist, and pedometer) to evaluate Oura EE variables and step count.

**Results:**

Strong correlations were shown for Oura versus indirect calorimetry in the laboratory (*r* = 0.93), and versus reference monitors for all variables in free-living (*r* ≥ 0.76). Significant (*p* < 0.05) mean differences for Oura versus reference methods were found for laboratory measured sitting (− 0.12 ± 0.28 MET), standing (− 0.27 ± 0.33 MET), fast walk (− 0.82 ± 1.92 MET) and very fast run (− 3.49 ± 3.94 MET), and for free-living step-count (2124 ± 4256 steps) and EE variables (MET: − 0.34-0.26; TEE: 362–494 kcal; AEE: − 487-259 kcal). In the laboratory, Oura tended to underestimate EE with increasing discrepancy as intensity increased.

**Conclusion:**

This is the first study investigating the validity of Oura Ring EE against gold standard methods. Oura successfully identified major changes between activities and/or intensities but was less responsive to detailed deviations within activities. In free-living, Oura step-count and EE variables tightly correlated with reference monitors, though with systemic over- or underestimations indicating somewhat low intra-individual validity of the ring versus the reference monitors. However, the correlations between the devices were high, suggesting that the Oura can detect differences at group-level for active and total energy expenditure, as well as step count.

## Background

Consumer-based activity trackers are increasingly used in research for measuring cardiac activity, such as heart rate (HR), heart rate variability (HRV), physical activity (PA) (e.g. steps, activity intensities, energy expenditure (EE)), and sleep (e.g. sleep stages and duration) [1–5]. Wearable devices reduces participation burden, thus improves adherence, compared to self-report methods such as PA logbooks [6]. The commercial devices have technologically advanced and increasingly improved comfortability and lightweight [7], and compared with research-grade accelerometers, are often less intrusive, cheaper, with improved storage and battery capacity [6, 8]. The unobtrusiveness and comfortability facilitate for convenient accumulation of large-scale, continuous, and long-term monitoring [7, 9, 10], subsequently providing novel opportunities for researchers and healthcare practitioners [7, 11]. Furthermore, long-term trends and day-to-day variations can present comprehensive insights into individuals’ health-status [7, 12]. For instance, alcohol consumption, pregnancy, fever, sleep disorders, or heavy exercise elevate mean nocturnal HR. [7] Accordingly, wearable devices may be used as a cost-effective instrument for quantification of physiological measurements in research and healthcare [7].

The Oura Ring is a new commercial multisensory wearable device that is lightweight (4–6 g) and operates for 5–7 consecutive days with one battery charge [7, 8, 11, 13]. With its subtle design, it may sometimes be preferred to more bulky activity watches or accelerometers. For physiological measurements, the ring utilises gyroscope and triaxial acceleration data, photoplethysmogram (PPG) signal, body temperature, and user’s body metrics (sex, age, body mass, height). PPG is a non-invasive optical technology that measures cyclical oscillations of blood circulation by emitting light on the skin and absorbing the light reflection through a light detector [8, 14, 15]. The ring quantifies PA (low, moderate, and high activity, inactivity, step count, MET, active EE (AEE), total EE (TEE)), body temperature, respiration rate, HR, HRV, and sleep [7, 11]. Sleep metrics [2, 7, 16], and nocturnal HR and HRV [6, 7] have been independently validated, with high accuracy and agreement in laboratory setting. Likewise, the ring has displayed promising results in monitoring the menstrual cycle based on night skin temperature [17], and been tested in predicting depression and anxiety symptoms [18], and in detecting COVID-19 [19]. Measurement of physical activity (i.e., EE) is important in healthcare research where PA is highly associated with health parameters, and accurate measurements are imperative. However, of all the PA and EE variables Oura offers, only the validity of step count, TEE, and sedentary time have been investigated [6, 20]. Those studies were only performed with ActiGraph reference monitors in free-living over 4.5 [20] or 7 days [6] with participants recruited through convenience sampling with either omitted or undefined daily PA. In addition to not validating the Oura variables with gold-standard methods, they used a narrow frequency filter (ActiGraph filter 0.29–1.63 Hz [21, 22]) for the reference methods in free-living. Although Henriksen, Svartdal [6] found strong correlations between Oura and the reference monitor for TEE and step count, measurement error and limits of agreement (LoA) were high, which was in line with Niela-Vilen, Azimi [20] findings for step count. Accordingly, the Oura Ring has potential to be used in research and healthcare. However, each variable needs to be independently validated against gold standard and reference methods in both laboratory and free-living setting in a diverse population sample for generalisation of the findings. Subsequently, that would allow for validation in both a controlled environment with well-defined workloads and highly reliable and valid equipment, as well as in a habitual setting addressing ecological validity.

The present study was designed to determine the validity of Oura Ring output. The first aim in this study sought to evaluate the accuracy of Oura Ring EE output compared with indirect calorimetry (IC) in laboratory setting. The second aim sought to evaluate the validity of Oura Ring EE variables and step count against reference monitors in free-living.

## Methods

### Study design

A study within the methodological project Measuring Energy expenditure and Diary intake at different Activity Levels (MEDAL) was performed to assess Oura Ring step count and EE measures and consisted of two parts: (1) a laboratory part where structured activities were performed while measuring oxygen consumption (VO_2_) and wearing the Oura Ring and three accelerometers positioned on the hip, thigh, and wrist; (2) a free-living part where participants wore the Oura Ring, pedometer, and accelerometers (hip, thigh, wrist) while conducting their life as normal. The laboratory part was used to validate Oura EE during different activities and intensities against IC, and for developing calibration models to predict EE for accelerometer output in free-living. Recruitment occurred between November and April 2021/22 in southwest Sweden.

### Participants and recruitment

Thirty-two low and highly active participants (17 females, 15 males) were recruited through advertising on university billboards, social media, and via contacts in sport clubs. While the final sample size was similar to other Oura Ring validation studies [6, 8, 11, 16, 20, 23], the MEDAL project initially aimed to recruit 40 participants but had to accept a slightly smaller sample size due to a limited time-period for data collection and access to devices needed for measurement. The study protocol and selection criteria of participants were designed to recruit a sample with large variation in PA level to enable device validation across the whole intensity span in both controlled and normal environments. Inclusion criteria comprised of compliance to study-protocol, ability to run for 4 min at 8 km·h^−1^ (determined through a direct question as a part of the booking of the participant for the laboratory measurement), and aged between 20 and 40 years. Self-reported vigorous PA (equivalent to running or ball/team sports) had to be < 150 min/week (excluding walking) or > 300 min/week for inclusion in a low, respective, a high active group. Exclusion criteria consisted of: (1) individuals with PA mainly involving cycling, swimming, or strength training, (2) medical conditions affecting resting metabolic rate (RMR) or compliance to study protocol, for example untreated/poorly regulated hypo- or hyperthyroidism, diabetes, cardiac diseases, active/post COVID-19 symptoms, (3) pacemaker, artificial joints, or metal elements bilaterally in the body, (4) current/attempting pregnancy, (5) otherwise considered, by the researchers, unsuitable for the study.

### Data collection

#### Laboratory setting

Laboratory measurements were conducted in the morning (either at 08:00 or 09:30), with participants informed to fast overnight and not to exercise the same day prior to the visit. Body mass and height were measured, and other participant characteristics (age, sex, self-reported PA frequency) collected before RMR measures (canopy with continuous airflow, lying quietly on a bed for 20 min, last 10 min used for RMR calculations) [24] followed by sensor fitting. The participant then performed five different activities according to a structured protocol while VO_2_ was recorded. RMR and VO_2_ during activity were recorded using a stationary metabolic system (Oxycon Pro, Jaeger, BD Corporation, Franklin Lakes, NJ, USA). To achieve VO_2_ steady state, each activity lasted for 4 min, using the last 2 min for EE calculation [25]. The activities included sitting, standing, standing-arranging books, walking (slow (4 km·h^−1^), fast (6 km·h^−1^)), and running (slow (9 km·h^−1^), fast (12 km·h^−1^), very fast (15 km·h^−1^), or until voluntary exhaustion). They were conducted indoors in a controlled laboratory with locomotive activities performed on a treadmill (RL2500E, Rodby, Vänge, Sweden). Metabolic equivalent of task (MET) was determined by the quotient of total activity-specific VO_2_ relative to the RMR VO_2_ [9, 21].

#### Free-living

Free-living data collection lasted for 2 weeks with participants wearing the Oura Ring, three accelerometers, and pedometer for the whole period, which is the longest study period, to date, for Oura Ring validation [2, 6–8, 11, 16, 20, 23]. The accelerometers were used in the laboratory to develop new prediction equations for EE in the free-living setting. The r^2^ prediction models in the laboratory were 0.932, 0.925 and 0.901 for the hip, thigh, and wrist respectively. The wearable devices were positioned identically as in the laboratory setting. All accelerometers were changed after 1 week of measurement for all participants to ensure sufficient battery and memory. All devices, but the pedometer, were waterproof. Participants were blinded to the data, except for pedometer step count. A short background questionnaire was completed at the initial visit. The participants were asked to document their sleep (bedtime, wake-up time), PA (duration, type), and device removal (time, duration) during the study period on structured self-report daily logbooks. The self-reported data were used to interpret and mitigate possible errors in the recorded data [8, 11]. Oral and written instructions and guidelines for device usage were provided.

### Oura ring

The Oura Ring (Gen 2, firmware 4.0.4, Oura oy, Finland) utilise gyroscope and triaxial acceleration data, PPG signal (250 Hz), body temperature, and user’s body metrics (sex, age, body mass, and height) to determine HR, HRV, respiratory rate, sleep parameters, EE, and PA [11, 23]. The Ring provides various health-related and well-being parameters, such as estimated mean and minute-by-minute METs, AEE, TEE, PA, step count, rest duration, non-wear time, and sleep duration [8]. Oura estimates respiratory rate at 30 s resolutions, MET at 60 s resolutions, and HR, HRV, and sleep stages at 5 min resolutions [26]. Step count is provided by Oura as a daily summary of total steps. MET [27] is the main unit of EE, and is used for aerobic exercise intensity categorisation where AEE is defined and starts accumulating at > 1.5 MET [23, 28]. The data was transferred and stored after the study period, via Bluetooth, to an Oura mobile app and cloud server, where the data later was extracted from for analysis [13]. The Oura ring was fitted and worn on self-selected fingers (excluding thumb).

### Accelerometers & pedometer

PA was measured by Axivity AX3 (Axivity AX3, Axivity Ltd., Newcastle upon Tyne, UK), which is a waterproof (to 1.5 m) triaxial accelerometer, equipped with temperature sensors, capturing acceleration along three orthogonal axes, can be positioned at various body locations [5], and considered feasible and practical for PA measures [5, 10]. The AX3 were set to capture acceleration at a sample rate of 100 Hz and a range of **±**8 g (where 1 g is equivalent to Earth’s gravity) [21]. The accelerometer locations examined in the laboratory and free-living were wrist, hip, and thigh, which are the most commonly used positions for accelerometers in physical activity research [29]. Oura was compared to the different positions since the association could differ depending on body position. For example, the wrist position is similar to the position of the Oura ring, thus could capture and generate similar data, while the hip position is the most used accelerometer position. Wrist and hip accelerometers were attached to elastic bands placed on the non-dominant dorsal wrist and laterally above the right hip, respectively, while the thigh accelerometer was attached by medical-grade adhesive film to the mid right anterior thigh. These placements are commonly used in epidemiological and clinical research [1, 29]. The pedometer (Yamax SW200 Digi-walker, Tokyo, Japan), which is an accurate, cost-effective, and simple way of monitoring step count [30, 31], was positioned on the right hip, aligned as an extended line from right ankle and knee, inside the hip accelerometer.

### Data analysis

This study investigated the accuracy of Oura MET in the laboratory against indirect calorimetry, and Oura MET, TEE, AEE, and steps count against reference monitors (3 accelerometers and pedometer) in free-living. Data analysis included the EE variables and step count extraction from the gathered data and statistical analysis leveraged to examine Oura Ring validity. Accelerometer AEE output was defined as > 1.5 MET to correspond to Oura AEE. The accelerometer output was time synchronised to the Oura ring, which reporting period starts and ends at 4 am each morning. Only valid days, defined at > 10 h of simultaneous wear-time while awake of the ring and reference monitors [4, 20], were included in the analysis. Non-wear time was defined as 60 min of zero accelerometer output after processing, with allowance of up to 2 min of interruptions below the sedentary threshold [32]. Participants were initially recruited into groups based on PA level but were combined as one group for analysis due to unbalanced groups.

**Fig. 1:**
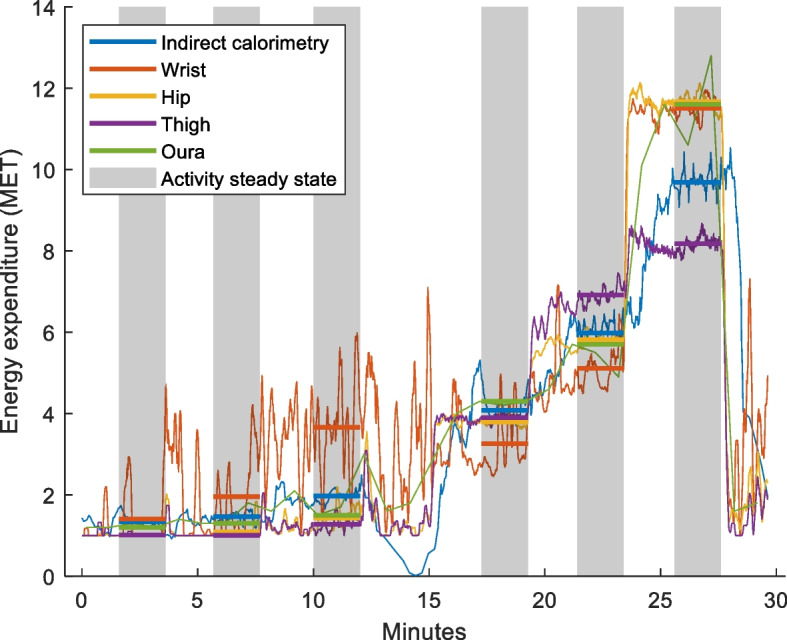
Example of raw data from laboratory setting

Figure 1 illustrates an example of raw data gathered from the laboratory setting. Raw accelerometer data was extracted with OmGUI software (Axivity Ltd., Newcastle upon Tyne, UK) and filtered using frequency extended method (FEM, 0.29-10 Hz) [9, 21, 22], which is a wider filter and shown to outperform the original actigraph filter (0.29–1.63 Hz) [21, 22]. Linear regression was used for calibration between filtered accelerometer output and measured EE in the laboratory. The regression model was then applied to the filtered free-living accelerometer data for EE estimation. Processing of accelerometer data and synchronisation with Oura data was performed in MATLAB 2021a (MathWorks, Natick, MA, USA). The statistical analysis was done using SPSS version 28 (IBM SPSS Statistics, Armonk, NY).

### Statistical analysis

Descriptive data are presented as mean ± standard deviation (SD) with statistical significance alpha level set to < 5%. Paired sample t-test was used for determination of bias [7, 20]. Within-individual correlations (r) were performed on pairwise EE variables and step count and then calculated as group mean (±SD) to assess the overall relationship between the Oura versus the IC (laboratory) and Oura versus the reference monitors (free-living) [7, 8, 20, 33]. Activity-by-activity correlation analysis were not performed since the structured protocol performed was designed to control both activities and intensities/velocities, thus not expecting large between-individual-variation. Correlation effect size was interpreted as: r > 0.1 small; > 0.3 moderate; > 0.5 large [33]. Mean absolute percentage error (MAPE) was calculated for the assessment of the size of the individual measurement error (%), with 10% cut-off for indication of low measurement error in free-living [6, 34]. Finally, model agreement was visualised by Bland-Altman plots [35].

### Ethical considerations

The study protocol was granted by the Regional Ethical Review Board in Gothenburg (Dnr 2019–05316, Dnr 2020–00010) and performed according to the ethical principles of the Declaration of Helsinki. All participants received oral and written information regarding the study and signed informed written consent and health declaration before enrolment. Participation was voluntary, with the right to withdraw at any time and without giving any reason. Participants received one cinema ticket (100SEK) upon monitoring completion and returning of devices, for compensation of participation.

## Results

**Fig. 2:**
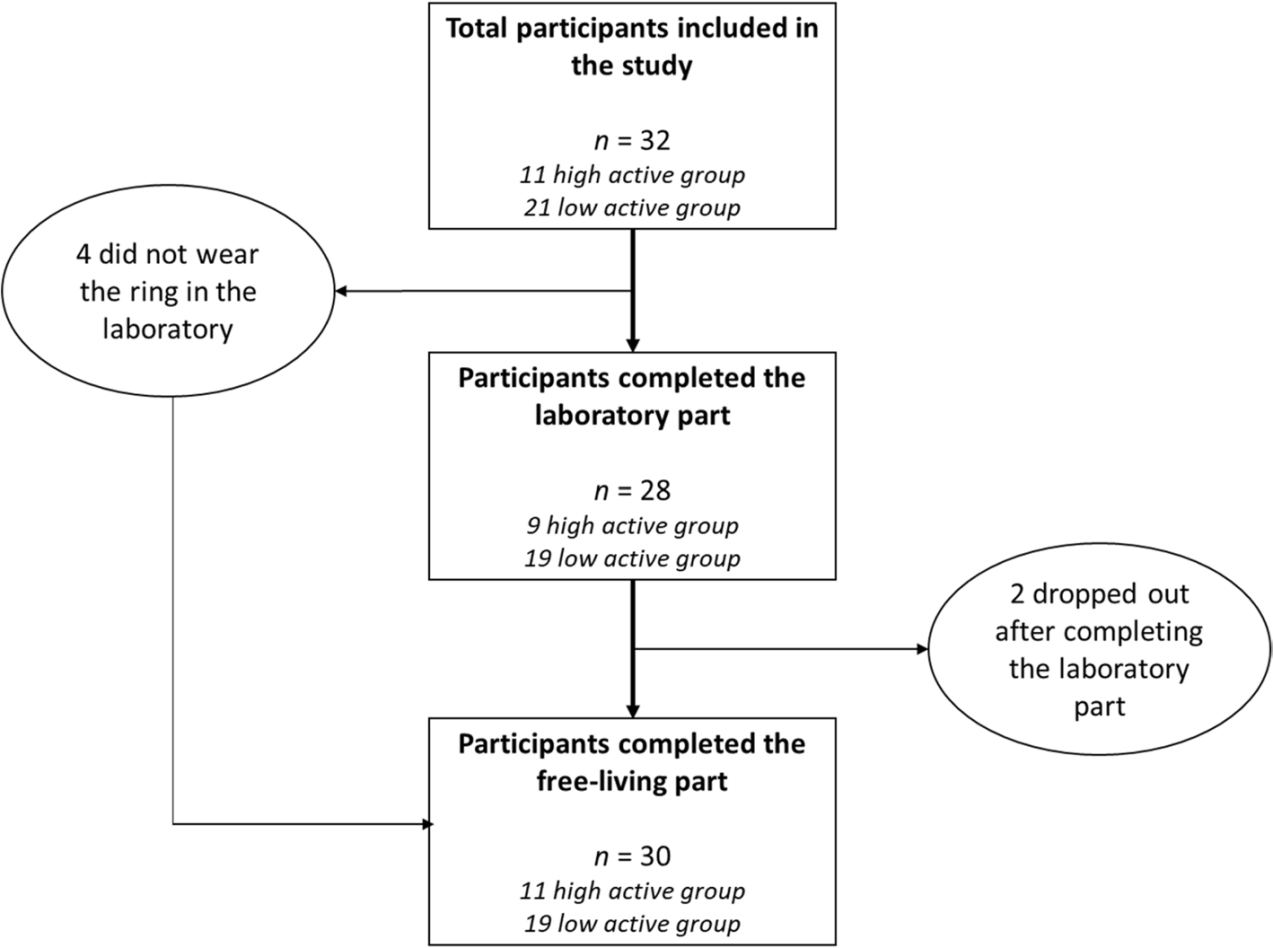
Study flowchart

Summary statistics for participant characteristics are presented in Table 1. Each participant had 5–14 (mean 13,1) valid days of free-living recording, totalling 393 valid person-days of simultaneous Oura, pedometer, and accelerometer usage. Of the 32 participants, 2 dropped out after completing the laboratory part, and 4 did not wear the ring in the laboratory but participated in the free-living part (Fig. 2). Thus, the total number of participants was 28 and 30 for the laboratory and free-living, respectively.

**Table 1:** Participant characteristics, mean ± SD

	Total	± SD
*n*	32	
PA/week^a^ (hrs)	4.3	4.9
Age (years)	29.6	5.2
Females (%)	53%	
Height (cm)	175.4	11.6
Body Mass (kg)	73.9	17.8
BMI (kg/m2)	24.0	5.0
RMR (kcal)	1485	326

*BMI* Body Mass Index, *PA* Physical Activity, *RMR* Resting Metabolic Rate

^a^Self-reported

**Fig. 3:**
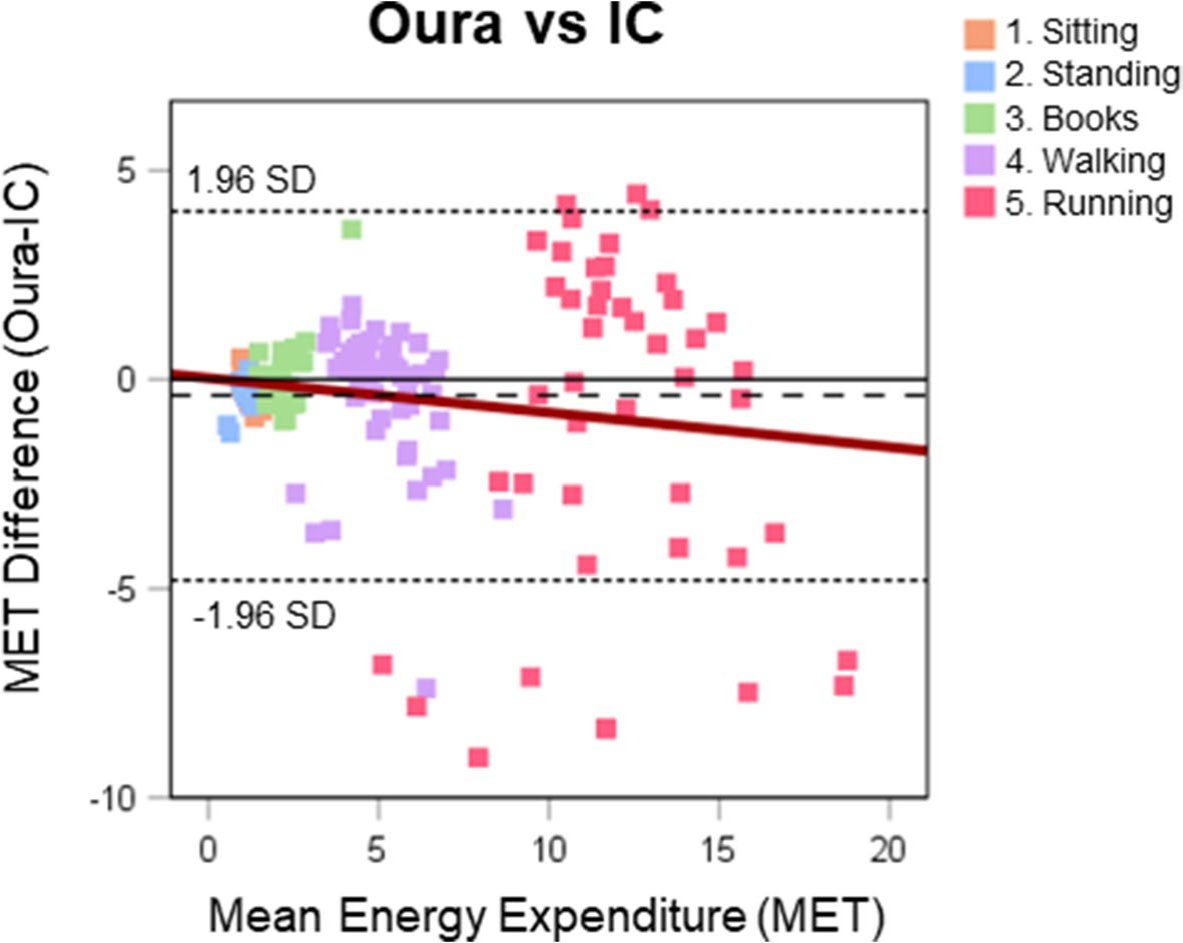
Comparison of Oura- and IC-derived energy expenditure metrics illustrated in a Bland-Altman plot. Squares depict the Oura and IC values for each activity performed in the laboratory by the participants. Dashed line depicts mean difference and dotted lines limits of agreement (±1.96 SD). IC, Indirect Calorimetry; MET, Metabolic Equivalent of Task

**Fig. 4:**
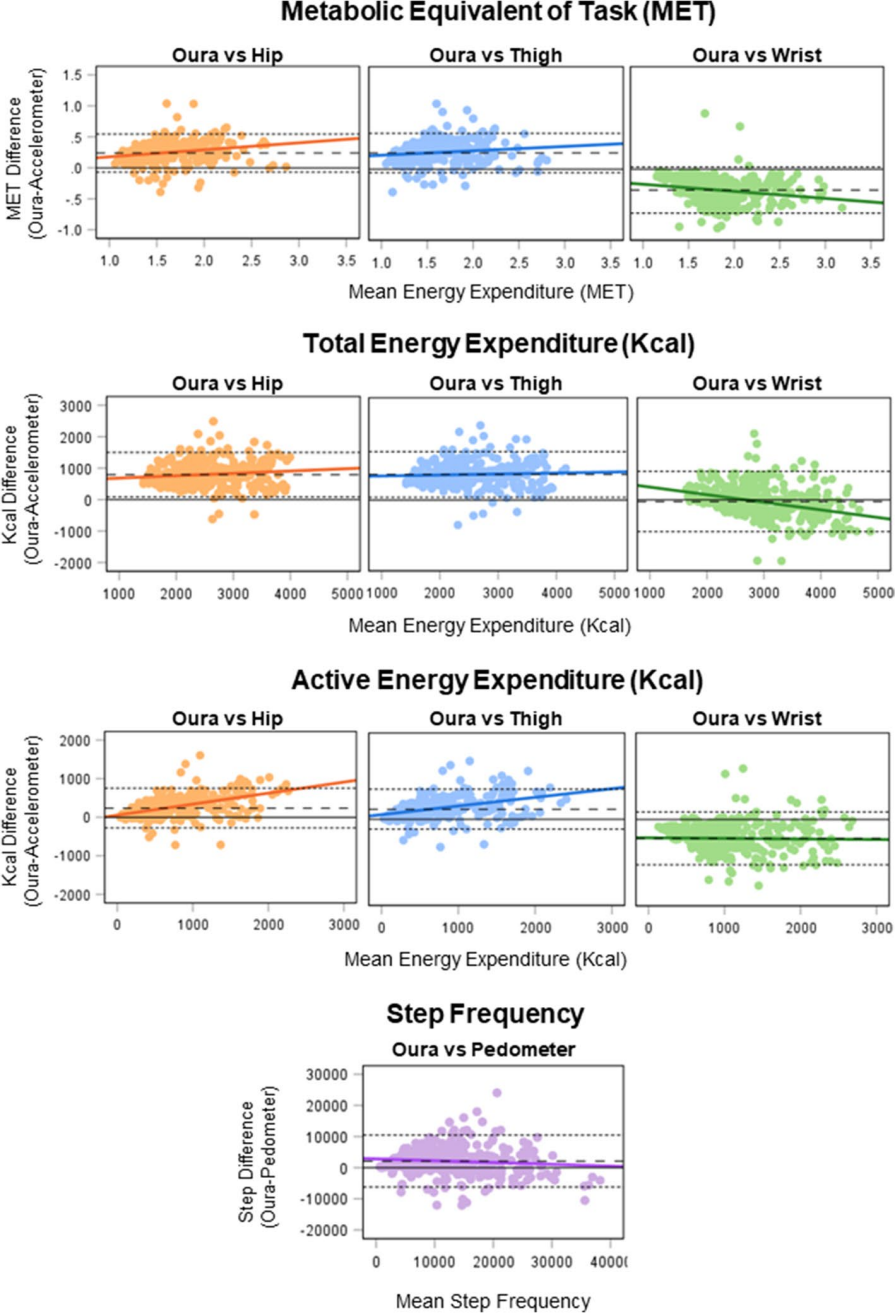
Comparison of free-living Oura- and accelerometer-derived energy expenditure metrics, and Oura- and pedometer-derived step metrics illustrated in Bland-Altman plots. Points depict the Oura and accelerometer or pedometer values for each day of data within the free-living setting. Dashed lines depict mean difference and dotted lines limits of agreement (±1.96 SD). IC, Indirect Calorimetry; Kcal, Kilo calories; MET, Metabolic Equivalent of Task

Table 2 outlines laboratory and free-living comparisons and are visualised in Figs. 3 and 4, respectively. There was a large mean individual correlation between Oura and IC for the laboratory activities, with small underestimation (− 0.4 MET) but with a MAPE of 21.1%. The bias was close to ideal and with relatively narrow LoA. For the activity-by-activity analysis, Oura tended to underestimate EE compared with IC, with significant differences seen for sitting, standing, fast walk, and very-fast run. The measurement error was high for the stationary activities and there was increasing measurement error with increasing walking and running intensities. These differences are visualised in the Bland-Altman plot (Fig. 3).

**Table 2:** Mean output (±SD), mean individual correlation (±SD), mean bias (±SD), 95% limits of agreement, and measurement error (MAPE) between Oura and IC energy expenditure (MET) in laboratory setting overall and activity-by-activity, and between Oura- and accelerometer-derived EE metrics, and Oura- and pedometer-derived step metrics in the free-living setting

			MET output		Correlation ***r***		Bias					Error
Activity	Sensor	***n***	Mean	±SD	Mean	±SD	Mean	±SD	***p***	95% Limits of Agreement		MAPE
										Upper	Lower	
**Laboratory**												
Combined activities	Oura	*183*	5.0	4.4	0.93	0.14	−0.4	2.25	0.02	4.03	−4.79	21.1%
	IC	*183*	5.4	4.8								
1.Sitting	Oura	28	1.13	0.13			−0.12	0.28	0.04	0.44	−0.67	6.6%
	IC	28	1.25	0.26								
2.Standing	Oura	28	1.06	0.35			−0.27	0.33	< 0.001	0.374	−0.921	16.3%
	IC	28	1.34	0.20								
3.Books	Oura	28	2.30	0.88			0.11	0.85	0.48	1.78	−1.56	24.1%
	IC	28	2.18	0.41								
4.Walk – slow	Oura	28	4.38	0.73			0.18	0.97	0.35	2.07	−1.72	18.8%
	IC	28	4.20	0.82								
4.Walk – fast	Oura	28	5.41	1.38			−0.82	1.92	0.03	2.93	−4.57	19.3%
	IC	28	6.23	1.46								
5.Run – slow	Oura	26	10.48	3.49			−0.17	3.81	0.83	7.29	−7.62	29.9%
	IC	26	10.65	1.83								
5.Run – fast	Oura	10	12.49	3.19			−1.11	4.35	0.44	7.42	−9.65	25.7%
	IC	10	13.60	2.76								
5.Run – very fast	Oura	9	13.70	2.69			−3.49	3.94	0.03	4.23	−11.21	20.7%
	IC	9	17.19	3.27								
**Free-Living**												
MET	Oura	389	1.66	0.35								
	Hip	389	1.42	0.32	0.79	0.29	0.24	0.16	< 0.001	0.55	−0.07	18.2%
	Thigh	393	1.40	0.33	0.81	0.19	0.26	0.16	< 0.001	0.57	−0.06	20.2%
	Wrist	388	1.99	0.39	0.80	0.24	−0.34	0.19	< 0.001	0.03	−0.71	17.2%
TEE (Kcal)	Oura	389	2883	637								
	Hip	389	2087	595	0.79	0.25	796	362	< 0.001	1506	85	42.2%
	Thigh	393	2066	621	0.78	0.23	817	375	< 0.001	1551	82	44.7%
	Wrist	388	2932	794	0.76	0.26	− 54	494	0.03	913	− 1022	13.0%
AEE (Kcal)	Oura	389	760	554								
	Hip	389	523	422	0.81	0.26	238	262	< 0.001	751	− 276	68.1%
	Thigh	393	500	451	0.81	0.20	259	263	< 0.001	774	− 255	89.5%
	Wrist	388	1241	562	0.79	0.24	−487	346	< 0.001	192	− 1166	46.2%
Steps	Oura	389	13,446	7374								
	Pedometer	389	11,322	7779	0.77	0.29	2124	4256	< 0.001	10,466	− 6217	50.3%

Significance determined with paired t-test

AEE Active Energy Expenditure, IC Indirect Calorimetry, Kcal Kilocalories, MAPE Mean Absolute Percentage Error, MET Metabolic Equivalent of Task, TEE Total Energy Expenditure

Large correlations were found for all comparisons between the Oura and reference monitors in free-living, with a close to ideal mean bias between Oura and wrist TEE (Table 2). Oura EE output was significantly different for all accelerometer comparisons, with relatively large LoA and tendencies to overestimate hip and thigh EE, and step count, while underestimating wrist EE. MAPE for free-living comparisons was only close to acceptable limits (13.0%) for wrist TEE.

## Discussion

### Main findings

The present study was designed to determine the validity of Oura Ring output. The first aim in this study sought to evaluate the accuracy of Oura Ring EE output compared with IC in laboratory setting. The second aim sought to evaluate the reliability of Oura Ring EE variables and step count against reference monitors in free-living. The results from the laboratory showed that the Oura successfully identified major changes between various activities and/or intensities (high individual correlation), but was less responsive to detailed deviations within different activities and/or intensities. Oura Ring step count and all EE variables correlated strongly with the reference monitors in free-living. Overall, the MET metric estimated by Oura ring corresponded best with the reference monitors.

### Laboratory setting

To our knowledge, this is the first study investigating the validity of Oura Ring EE output in laboratory setting. The most noticeable was the strong individual correlation with small mean bias thus, successfully identified major changes between various activities and/or intensities. However, the ring was less responsive to detailed deviations within different activities and/or intensities, with significant underestimation for the stationary activities. Additionally, in line with previous finding in commercial activity trackers, Oura tended to underestimate EE compared with the IC with a greater discrepancy as intensity increased, with increasing individual measurement error [36–38]. EE during activity has been suggested to be the most unpredictable and variable component of TEE [39, 40]. Nevertheless, accuracy and validity of EE output in commercial devices have increased over the years [6, 38], however, the mean difference and measurement error for Oura Ring EE variables in the current study were greater than previously seen for Oura HR, HRV, and sleep metrics, suggesting accuracy can be improved further [3, 7, 38].

### Free-living

#### Energy expenditure

In free-living, all Oura Ring EE variables strongly correlated with the reference monitors. Oura TEE versus wrist presented a strong correlation, agreement, and almost perfect mean bias. Surprisingly, Oura showed overall similar correspondence with the 3 accelerometers despite different placements on the body, which was unexpected, due to the ring and wrist positions resemblance. However, Fridolfsson, Arvidsson [1] have previously shown hip and thigh positions to correlate stronger with measured EE compared to the wrist. Other studies have suggested that the hip position consistently outperform the wrist which, in turn, seem to be superior to thigh [9, 37]. Nevertheless, no previous studies have investigated the accuracy of Oura AEE output, and the strong correlations indicate a reliable measure at group level of Oura AEE output compared with the accelerometers. Estimation of energy cost during activities have been shown to be the most unpredictable and variable component of TEE due to the great movement variation of PA [39, 40]. Moreover, Oura defines AEE as PA intensities exceeding 1.5 MET [28], whereas traditionally in research AEE starts accumulating 1.0 MET. The delimitation of AEE as ≥1.5 MET may be set because sedentary activity is usually recognised between 1.0–1.5 MET, while AEE often is used for measuring health behaviours. Similar to discoveries for commercial activity watches [40], Oura systematically over- or underestimated most EE variables compared with the accelerometers. Compared with the only previous study examining Oura TEE validity, the current study found a stronger correlation (*r* 0.79 vs *r* 0.70), similar measurement error (MAPE 13% vs 13%), and LoA range (85–1506 vs − 624 – 920), but larger mean bias (796 vs 148) [6] for Oura TEE compared with hip-placed accelerometers. However, the comparison with the previous study results may be limited by large variation in methodology. Since Henriksen, Svartdal [6] publication, Oura soft- and firmware have been updated, and while they used a hip-placed accelerometer, they used a different reference monitor and had less valid days which can influence MAPE. Ours is the first study examining validity of Oura MET output. Although not identical, MET and TEE output are calculated with both resting and active EE [1, 9, 27], thus generate similar information. The main proportion of TEE is usually expended during low intensity levels or at rest [39], thus, the accuracy of estimated RMR (accuracy of inputted data and equations, and the variables used for calculation) likely influence the findings for MET and TEE. Therefore, it is plausible that the measured RMR in the current study is lower than the calculated Oura RMR, which likely is based on sex, age, body mass, and height, but not disclosed by the manufacturer.

#### Steps

The Oura Ring step count correlated strongly with the pedometer. Meanwhile, Oura significantly overestimated step count, which have also been stated in two previous studies comparing Oura to hip- [6] or wrist-worn [20] accelerometers in free-living. The previous studies also found Oura step count to correlate strongly with the reference monitor. Moreover, Niela-Vilen, Azimi [20] reported smaller mean difference but smaller measurement error (1416, MAPE 5.2%) as the current study, while Henriksen, Svartdal [6] presented higher values (3779, MAPE 69%). The smaller mean bias identified by Niela-Vilen, Azimi [20] may be attributed to Oura and reference monitor being worn on the same hand, compared with hip-worn and/or self-selected finger as by Henriksen, Svartdal [6] and the present study. Likewise, a 2020 review on step count accuracy for consumer-based activity trackers found heterogeneity of over- and underestimation between and within brands [38]. They ascribed the observed variability to differences in wear locations and number of comparisons for each sensor.

### Strength & limitations

A major strength in the study was that gold-standard criterion was used for the laboratory comparisons [39, 40]. Additionally, we investigated the Oura Ring in two conditions; (1) controlled laboratory setting, and (2) free-living (ecological validity) (3), against participant with low and high daily PA level to allow for device validation across the whole intensity span in both controlled and normal environments. Furthermore, multiple days of recordings was included for each participant [6], with simultaneous worn sensors for up to 14 days, which is the longest study period for Oura Ring validation [2, 6–8, 11, 16, 20, 23]. Sample size was similar to other Oura Ring validation studies [6, 8, 11, 16, 20, 23], with an even gender balance and varied level of PA, providing a diverse sample [20].

Limitations of the study includes the usage of non-gold standard criteria in free-living [39, 40], Oura Ring placement on self-selected fingers instead of on the non-dominant hand as the wrist accelerometer [20, 41], small sample size performing the fast running activities, and that only healthy participants, with a relatively small range in age and BMI, were included in our study which may limit the generalisability of the findings [11, 20].

## Conclusion

In the laboratory, the Oura Ring successfully identified major changes in PA with overall small measurement error but was less responsive to detailed deviations, with increasing discrepancy along with increases in intensity. In free-living, Oura step count and all EE variables correlated strongly versus the reference monitors, but often with differences in means. The mean bias and measurement error seen for the ring in the present study were greater than for other validated Oura variables (HR, HRV, Sleep variables), suggesting potential to improve Oura EE accuracy. Accordingly, the Oura Ring cannot unambiguously be recommended to be used interchangeably with the reference monitors in the study. Lastly, while some variables presented large limits of agreement, indicating somewhat low intra-individual validity of the ring versus the reference monitors, the correlations between the devices were high, suggesting that the Oura can present differences at group-level for active and total energy expenditure, and step count. Future work should include assessing Oura EE variables against gold standard methods in free-living of different population groups.

## Data Availability

The present dataset is available upon reasonable request. Requests for data sharing from appropriate researchers and entities will be considered on a case-by-case basis. Interested parties should contact the corresponding author or Gothenburg University legal officer erica.schweder@gu.se.

## Abbreviations

AEE: Active Energy Expenditure
BMI: Body Mass Index
EE: Energy Expenditure
FEM: Frequency Extended Method
HR: Heart Rate
HRV: Heart Rate Variability
IC: Indirect Calorimetry
LoA: Limits of Agreement
MET: Metabolic Equivalent of Task
MAPE: Mean Absolute Percentage Error
PA: Physical Activity
PPG: Photoplethysmograph/Photoplethysmogram
RMR: Resting Metabolic Rate
SD: Standard deviation
TEE: Total Energy expenditure
VO_2_: Oxygen Consumption
