# Correction: Validation of Oura ring energy expenditure and steps in laboratory and free‑living

**DOI:** 10.1186/s12874-023-02029-w

**Published:** 2023-09-09

**Authors:** Emilia Kristiansson, Jonatan Fridolfsson, Daniel Arvidsson, Agneta Holmäng, Mats Börjesson, Ulrika Andersson‑Hall

**Affiliations:** 1 Center for Health and Performance, Department of Food and Nutrition, and Sport Science Faculty of Education, University of Gothenburg, Gothenburg, Sweden; 2 Institute of Neuroscience and Physiology, Department of Physiology, Sahlgrenska Academy, University of Gothenburg, Gothenburg, Sweden; 3 Department of Molecular and Clinical Medicine, Institute of Medicine, Sahlgrenska Academy, University of Gothenburg, Gothenburg, Sweden; 4 Sahlgrenska University Hospital, Region Västra Götaland, Gothenburg, Sweden

**Correction: BMC Medical Research Methodology 23, 50 (2023)**

10.1186/s12874-023-01868-x

Following the publication of the original article [1], the authors requested to update the MAPE values in Table 2, and the text that is linked to the new MAPE values.

The original article [1] has been updated.
